# Unhealthy diets, obesity and time discounting: a systematic literature review and network analysis

**DOI:** 10.1111/obr.12431

**Published:** 2016-06-03

**Authors:** Pepita Barlow, Aaron Reeves, Martin McKee, Gauden Galea, David Stuckler

**Affiliations:** ^1^ Department of SociologyUniversity of OxfordOxfordUK; ^2^ International Inequalities InstituteLondon School of EconomicsLondonUK; ^3^ Department of Public Health and PolicyLondon School of Hygiene and Tropical MedicineLondonUK; ^4^ Division of Noncommunicable Diseases and Life‐course, Regional Office for EuropeWorld Health OrganizationCopenhagenDenmark

**Keywords:** diets, obesity, overweight, time discount

## Abstract

There is an increasing policy commitment to address the avoidable burdens of unhealthy diet, overweight and obesity. However, to design effective policies, it is important to understand why people make unhealthy dietary choices. Research from behavioural economics suggests a critical role for time discounting, which describes how people's value of a reward, such as better health, decreases with delay to its receipt. We systematically reviewed the literature on the relationship of time discounting with unhealthy diets, overweight and obesity in Web of Science and PubMed. We identified 41 studies that met our inclusion criteria as they examined the association between time discount rates and (i) unhealthy food consumption; (ii) overweight and (iii) response to dietary and weight loss interventions. Nineteen out of 25 cross‐sectional studies found time discount rates positively associated with overweight, obesity and unhealthy diets. Experimental studies indicated that lower time discounting was associated with greater weight loss. Findings varied by how time discount rates were measured; stronger results were observed for food than monetary‐based measurements. Network co‐citation analysis revealed a concentration of research in nutrition journals. Overall, there is moderate evidence that high time discounting is a significant risk factor for unhealthy diets, overweight and obesity and may serve as an important target for intervention. © 2016 The Authors Obesity Reviews published by John Wiley & Sons Ltd on behalf of International Association for the Study of Obesity (IASO)

## Introduction

Unhealthy diets, overweight and obesity contribute to a number of chronic non‐communicable diseases and are among the leading risk factors for death and disability worldwide 1. Many policymakers have committed to promoting healthy diets and curtailing the growth of overweight and obesity 2. However, to design effective policy interventions, it is important to understand why people make unhealthy dietary choices 3.

Time discounting is a factor that is receiving growing attention as a potentially common cause of multiple risky behaviours 4. It describes how people value a reward to a lesser degree the farther in the future it is received 5. Given a choice, most people prefer smaller immediate rewards over larger rewards available after a delay – that is, they ‘discount’ the value of a reward in the future 6. Time discounting is a dimension of impulsivity in decision‐making and can be thought of as an index of an individual's cognitive ability to delay gratification. In turn, time discounting is linked to motivational processes by potentially explaining the inability to follow through with a specific behaviour despite an initial motivation to do so 7.

Time discounting varies considerably among individuals 8, 9, 10, tending to be higher among younger persons 11, 12, and in lower socioeconomic status 11 and less‐educated persons 13, 14 who are also at higher obesity risk 15. Thus, previous studies suggest that time discounting is a mechanism linking underlying environmental, social and life‐course factors to downstream risky unhealthy behaviours and associated health outcomes 16. This includes diets: the benefits of healthy diets may involve delaying gratification, whereas some unhealthy foods, such as sugar‐sweetened beverages, offer immediate rewards at the expense of long‐term harm 17. Researchers have therefore hypothesized that higher time discount rates could explain why some people are more likely to have unhealthy diets and respond unsuccessfully to interventions aimed at encouraging dietary change 18, 19, 20. Here, unhealthy diets include (i) high total caloric intake; (ii) high consumption of sugar and fat and (iii) low consumption of vegetables and fruits 20, 21, 22. Because unhealthy diets are linked to overweight or obesity, researchers also hypothesize that higher time discount rates are associated with being overweight or obese and poor response to weight loss interventions 23.

Over the past decade, a growing number of studies have found time discounting positively associated with unhealthy diets, overweight, obesity and binge eating disorder (BED) 18, 19, 20, 23, 24, 25. Yet, to our knowledge, previous reviews of this topic focussed on addictive or unhealthy behaviour in general 6, 7. Here, we conduct, to our knowledge for the first time, a systematic review of the evidence on time discounting and obesity, overweight and diets. Our study asks four main questions. First, is time discounting a risk factor for unhealthy diets, overweight and obesity and poor treatment response? Second, is this association modifiable by changing discount rates? Third, studies on time discounting draw on a range of methods for measuring time discount rates. We therefore also ask whether study findings are sensitive to discount rate measurement methods used. Fourth, theories of time discounting can integrate several disciplines, potentially including economics, psychology and social science 16. We therefore ask what are the disciplinary origins and patterns of cross‐disciplinary citation of studies in this field?

## Data and methods

### Search strategy and study selection

We searched Web of Science and PubMed for articles containing ‘time discounting’, ‘food’ and related terms, as described in Table 1. Searching across both databases enabled us to include studies published in journals from a range of disciplines, including economics, pharmacology and neuroscience. Both databases provide journal citation data that can be used for co‐citation analysis.

**Table 1:** Search terms

"time preference" diet
"time preferences" diet
"time‐preference" diet
"time‐preferences" diet
delay discount diet
delay discounting diet
delay‐discount diet
delay‐discounting diet
discount rate diet
inter temporal diet
inter‐temporal diet
intertemporal diet
time discount diet
time discounting diet
time‐discount diet
time‐discounting diet
"time preference" food
"time preferences" food
"time‐preference" food
"time‐preferences" food
delay discount food
delay discounting food
delay‐discount food
delay‐discounting food
discount rate food
inter temporal food
inter‐temporal food
intertemporal food
time discount food
time discounting food
time‐discount food
time‐discounting food
"time preference" obesity
"time preferences" obesity
"time‐preference" obesity
"time‐preferences" obesity
delay discount obesity
delay discounting obesity
delay‐discount obesity
delay‐discounting obesity
discount rate obesity
inter temporal obesity
inter‐temporal obesity
intertemporal obesity
time discount obesity
time discounting obesity
time‐discount obesity
time‐discounting obesity

**Figure 1:**
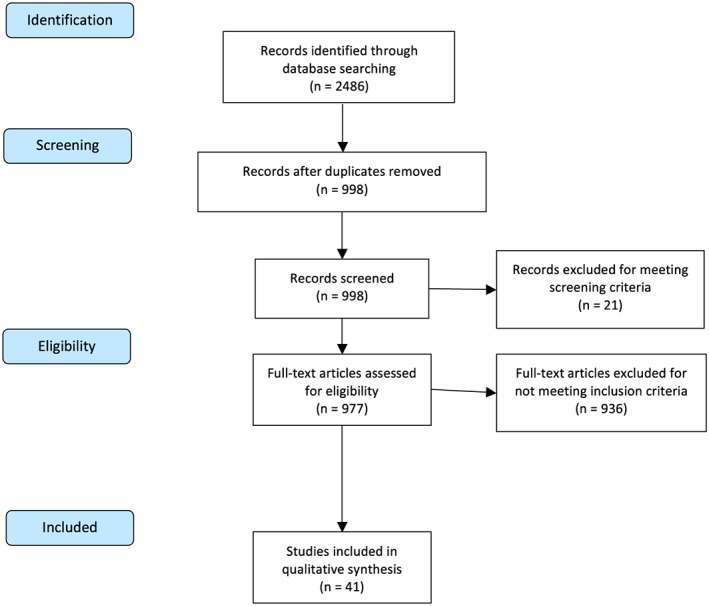
PRISMA flow diagram showing study identification, screening and exclusion. *Notes*: PRISMA flow diagram template from Moher *et al.* 2009 28.

We conducted our review according to the PRISMA statement for systematic reviews (see Web Appendix 1 for full checklist) 26. Figure 1 shows a PRISMA flow diagram depicting study identification, screening and exclusion. Our search on 22 June 2015 yielded 998 unique titles published from 1977 to mid‐2015. Papers were excluded if they were not in English or were not published articles. We included studies that examined the association between time discount rates and (i) unhealthy food consumption; (ii) overweight and obesity and (iii) response to interventions aimed at reducing unhealthy food consumption and body weight. Because of heterogeneity in study methodologies, we included studies that used a range of measurement methods, including computerized experimental tasks and survey self‐reports. Studies that did not meet our criteria were excluded. In total, 933 studies were excluded. Screening and exclusion were conducted by the lead reviewer (P. B.). Our final analytical sample included 41 studies, covering the years 2000 to 2015, although 40 of these studies were published since 2010.

### Data extraction and analysis

We extracted title, author, journal, abstract and year. Additional data were extracted from each paper and used to evaluate risk of bias, including research question, methodology, sample size, sample demographic, discounting measure, diet or weight‐related variables (e.g. body mass index (BMI), obesity and weight loss) and main results (Web Appendix 1). To assure consistency in study coding, a second author (A. R.) selected 10% of the papers at random and verified the suitability of each study's inclusion in our review and independently coded it, with any differences resolved by discussion. We performed a qualitative synthesis of study findings rather than conducting a meta‐analysis because there was heterogeneity between studies in the research question, research design and discount rate measures used.

Co‐citation data were collected from Web of Science and PubMed and analysed using VOSviewer version 1.6.1 and network clustering algorithms. These map the spatial location for each journal by minimizing the weighted sum of the squared Euclidean distances between all pairs of journal citations. Weights correspond to the strength of co‐citation, with higher values reflecting a greater tendency for journals to be cited together in the same article. Minimization of the distance between journals is subject to the constraint that the average distance between two items equals 1 27.

## Results

First, we describe trends, disciplinary patterns and methods used to measure discount parameters in the reviewed literature. Next, we describe the main findings by the type of studies of the association between time discounting and unhealthy diets, overweight and obesity, discount rate modifiers and treatment response. Finally, we describe results from studies using multiple discount rate measurement methods.

### Trends in and types of publications on time discounting and diets, overweight and obesity

**Figure 2:**
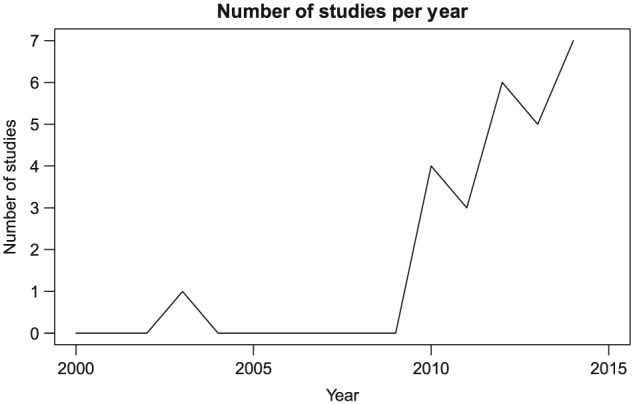
Number of food and discounting studies published per year, 2000–2014. *Notes*: The figure shows the number of studies published per year that was included in our review (see exclusion criteria in Table 1). Studies from 2015 were not included as search was conducted part way through the year. In 2015, 12 studies had been published by the date we searched PubMed and Web of Science (22/06/15).

Figure 2 plots the number of studies published per year that was included in our analysis. There is a marked increase in the number per year from 2010, after which the majority (93%) of studies in our analysis was published.

Among the 44 articles included in our review, 25 were cross‐sectional, 13 were experiments and three were longitudinal. Sample sizes ranged from 14 to 63,950. The mean sample size was 1748, albeit highly skewed to the right (median = 85). The majority of studies sourced their data via convenience sampling: 40 studies recruited voluntary participants via convenience sampling from local communities, of which 20 also applied sample quotas (i.e. targets for specific demographics), and four studies used large‐scale datasets with probability sampling. A large proportion of studies (14 out of 41) restricted their samples to women only, and 16 studies restricted their samples to children, adolescents and university students only (16 out of 44 studies).

### Disciplinary patterns of concentration and co‐citation

**Figure 3:**
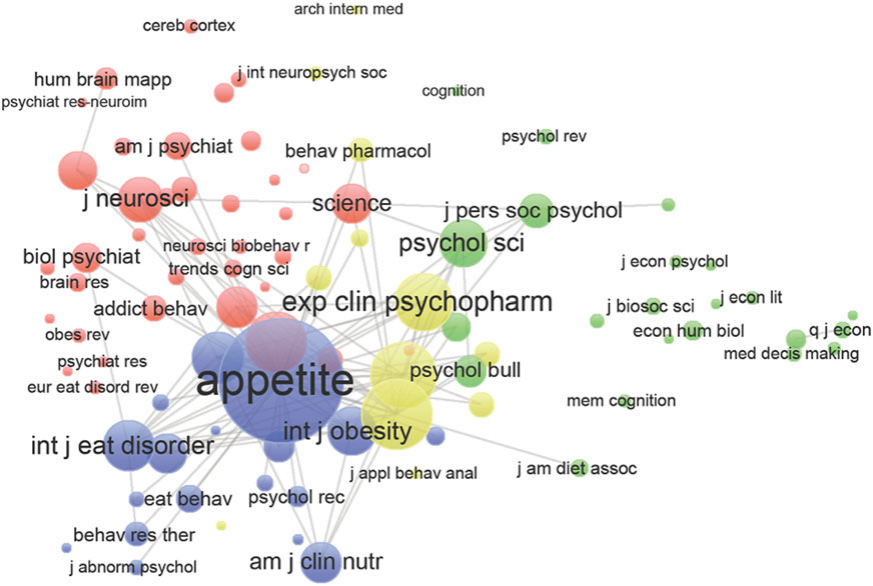
Co‐citation of journals. *Notes*: Bubble sizes correspond to the relative magnitude of each journal's citations in other journals (minimum five citations per journal; *n* = 90 journals). Proximity of bubbles corresponds to the frequency with which journals are cited together in other journals. The colours reflect communities identified by VOS clustering. Produced in VOSviewer Version 1.6.1.

The 41 studies were distributed across 22 journals. Half were published in two journals: *Appetite* (40.9%) and *Plos One* (9.1%). Figure 3 shows journal co‐citation patterns. The journals included in the co‐citation analysis are the 51 journals cited at least 10 times within the studies we reviewed.

As shown in Figure 3, four journal clusters are visible: nutrition, neuroscience, pharmacology and economic psychology disciplines. The most highly cited journal is *Appetite*. The nutrition cluster has a tendency for co‐citation with pharmacology and a slightly weaker tendency for co‐citation with neuroscience. The economic psychology cluster is the most isolated.

### Discount rate measurement

The methods used to measure time discounting varied in three main ways, and these are (i) how the survey was administered; (ii) how the discount rate was estimated and (iii) the nature of the rewards linked to the hypothetical choices.

Surveys can be administered in two ways, using a paper or online questionnaire or getting the subject to make choices as the options change incrementally on a computer. In both approaches, individuals state whether they would prefer a smaller reward now or a larger reward at some specified later time. The questionnaire method follows the ‘Monetary Choice Questionnaire’ method of Kirby *et al.*, in which choices vary by the size of reward and how long they must wait for the reward 28. In computerized tasks, respondents make choices on a computer between a series of immediate and delayed rewards but, unlike in the questionnaire, subsequent questions adjust the immediate reward amount by small increments, for example, by ±$0.50 29, until respondents state that they prefer it over the delayed reward (see, for example, Weller *et al.* 2008) 29.

In both methods, responses are then used to estimate discount rates. The first step is to determine an ‘indifference point’. This is the point at which respondents switch from preferring a delayed over immediate reward or vice versa. Respondents are said to subjectively value immediate and delayed rewards equivalently at the point at which they switch; that is, they are indifferent between the two 5, 30. The second step is to estimate discount rates from indifference points. The two main methods are (i) fitting a hyperbolic model or (ii) using an ‘area under the curve’ (AUC) method. In the hyperbolic method, discount rates are assumed to follow ‘dynamic inconsistency’ 10, 31. This refers to the commonly documented tendency to discount rewards steeply when choosing in the immediate and short run, resulting in a preference for the immediate reward. Rewards further into the future are discounted less steeply, increasing the tendency to prefer delayed rewards 10. Discount rates are estimated by fitting Mazur's (1987) hyperbolic model to a series of individual indifference points, as described in the following equation 32:

(1) $$Y=\frac{A}{\left(1+kx\right)}$$

where *Y* is the subjective value of a reward of amount A, *k* is the discount parameter and *x* is the delay to the reward's receipt 5. In contrast, the AUC method does not assume that discounting rates take a particular functional form. Instead, subjective values and associated delays to receipt from the discounting task or questionnaire are normalized and plotted, joining points with straight lines. The area between the plotted lines and the *x*‐axis is calculated, with smaller AUC values reflecting steeper discounting 5.

The third difference relates to the reward type. In 39 studies, the choices were purely hypothetical. They included hypothetical money (33 studies), food (34 studies) and weight loss (one study). Estimation of non‐monetary rewards followed a similar procedure to money: respondents chose between an amount of food or immediate weight loss and a larger, delayed reward of the same type. A second approach, used in two studies, is to encourage respondents to treat their responses as real by rolling a dice or selecting one response at random at the end of the survey and honouring it, so‐called ‘quasi‐real’ rewards.

### Systematic review findings: time discounting and unhealthy diets

Our search identified 25 cross‐sectional studies that analysed the association between time discounting and a number of diet and weight‐related variables including BMI 21, 23, 24, 33, 34, 35, 36, 37, 38, body weight 23, obesity 24, 25, 29, 39, 40, 41, 42, 43, 44, unhealthy diets and fast‐food consumption 21, 22, 45, 46, per cent body fat and binge eating 36, 47. Across all variables, 12 reported positive correlations with time discounting, seven reported varying results according to gender, time discount measure model specification and seven reported null findings.

Four studies analysed the association between time discounting and unhealthy diets.

Reslan *et al.* (2012) report higher demand and lower price elasticity of demand for high‐sugar and high‐fat foods among those with the highest discount rates in a sample of 21 female university students 46. A study by DeVoe *et al.* (2013) adopted an ecological approach, reporting higher discounting rates in US regions with more fast‐food restaurants whilst, in the same study, subjects randomly asked to recall experiences of fast food reported higher discount rates than controls who were not asked 48. Privitera *et al.* (2015) test for an association between time discounting for food and a 25‐item Disordered Eating Attitude Scale, with higher values on the scale representing more negative or disordered eating attitudes. The authors report lower discount rates among men with higher Disordered Eating Attitude Scale scores but find no association among women 35. Garza *et al.* (2013) report lower discounting rates among individuals with higher dietary quality (assessed by measuring fruit, vegetable, fat, milk and sugar‐sweetened beverage consumption), yet these differences were not significant at the *α* = 0.05 level once age, gender and education were accounted for 45.

### Time discounting, overweight and obesity

We identified two longitudinal studies that asked whether there were associations between weight change and changes delay discounting of hypothetical monetary rewards, reporting contrasting results 49, 50. First, Courtemanche *et al.* (2015) adopt an ecological approach to analyse the effects of food prices on BMI change. They document an interaction effect with time discounting, with lower prices correlated with greater BMI gains among those with higher discount rates 50. Second, Kishinevsky *et al.* (2012) collect functional magnetic resonance imaging data from 19 obese women whilst conducting a series of delay discounting tasks and find no association between discount rates and BMI or weight change over the subsequent 1.3–2.9 years. Yet individuals with less activation in areas of the brain associated with inhibitory control (inferior, middle and superior frontal gyri) during ‘easy’ discounting tasks were more likely to gain weight 49.

Five cross‐sectional studies report higher discount rates in individuals with higher BMIs when measuring discounting using hypothetical monetary 21, 23, 24, 38 and food rewards 35. Further, four studies reported higher delay discounting among obese compared with non‐obese individuals 24, 41, 42, 43, 44. Hendrickson *et al.* (2015) report higher discounting of hypothetical money and food in individuals wither higher per cent body fat 47. Turning to disordered eating, Manwaring *et al.* (2011) report higher discounting for food rewards among individuals suffering from BED compared with obese, non‐obese and non‐BED groups. The authors also report higher discount rates for food among obese compared with non‐obese and non‐BED controls. These differences were not significant when testing for differences in discounting for monetary rewards 51. Weller *et al.* (2008) report higher discounting in obese compared with healthy‐weight women, but find no difference in men 29. Davis *et al.* (2010) report higher discounting of monetary rewards in a group of obese individuals suffering from BED, but these differences lost significance once education was accounted for. Four studies reported null findings in testing for an association between discounting of hypothetical monetary rewards and BMI 33, 52, obesity 39 and binge eating disorder 37. One study also reported no association between healthy food consumption and discounting of both hypothetical food and monetary rewards 22.

### Discount rate modifiers

Eight studies identified significant effects of interventions aimed at modifying discount rates 19, 53, 54, 55, 56, 57, 58, 59, 60. Turning first to psychological modifiers, Hendrickson *et al.* (2013) compare the reduction in delay discounting among a sample of university students participating in a mindful eating workshop and those watching an educational video (the ‘control’ condition). They report greater reductions in delay discounting for food but not monetary rewards among those who participated in the workshop, compared with those who watched the video 53. Further, Daniel *et al.* (2013) analyse the effects of a prospective imagery intervention designed to induce increased thinking about the future. The authors compare discount rates for hypothetical monetary rewards in lean and obese individuals, finding no significant difference at the *α* = 0.05 level in the reduction in discount parameters in both groups 54. Neveu *et al.* (2014) find that carrying out a reasoning task after exposure to a food image cue reduced discount rates among participants with bulimia nervosa 59. Finally, Appelhans *et al.* and Rollins *et al.* report a positive association between the ‘relative reinforcing value of food’ and food consumption in a laboratory environment at high but not low discount rates 19, 20.

Two studies reported an effect of hunger on discount rates. De Ridder *et al.* (2014) compared participants randomly assigned to a ‘hungry condition’ where they were instructed to refrain from eating the previous evening and complete a discounting task before breakfast and sated participants who were first provided with breakfast. Those in the ‘hungry condition’ had lower discounting rates for large hypothetical monetary rewards compared with those in the sated condition 55. In contrast, Sellitto *et al.* (2014) test whether discount rates are modified by aversive cues. These are cues that are designed to stimulate a negative neural wave (error‐related negativity) that is associated with being better able to avoid events with harmful long‐term consequences. The authors hypothesized that time‐discount rates may be reduced by pairing aversive cues with foods (creating ‘error‐prone foods’) designed to stimulate increases in error‐related negativity. They find that combining foods with aversive cues decreased discount rates compared with foods that were not paired with aversive cues in sated participants; yet there was no variation among hungry participants 56.

The remaining two studies identified biological modifiers. In a sample of 87 adolescents, Lu *et al.* (2014) test whether cortisol reactivity mediates the association between delay discounting and per cent body fat. Cortisol is a hormone released in response to stress, improving the individual's capacity to respond to stress‐inducing situations and environments. However, cortisol also dampens the inflammatory response, which in the long term may increase oxidative damage. Cortisol reactivity is the responsiveness of cortisol levels to a given stimulus and, if excessive, may be harmful because of this impact on the inflammatory response. The authors report significant pairwise correlations in girls, with greater cortisol reactivity to stress associated with both higher discount parameters and higher per cent body fat 57. Wang and Dvorak (2010) test for between‐group and within‐subject changes in delay discounting of quasi‐real monetary rewards and blood glucose levels after being randomly assigned to a caffeine‐free soda either with or without sugar, finding lower discount rates among those assigned the sugary soda 58.

Three studies reported null findings. Leitch *et al.* (2013) find that subjects required to fast the night before a laboratory study did not report significantly different discount rates than those who could eat *ad libitum* 18. Kekic *et al.* (2014) test whether transcranial direct simulation modified discount rates and food cravings, finding no within‐subject differences in discount rates following real compared with sham stimulation, although food cravings for sweet foods were reduced 61.

### Time discounting and intervention response

Three studies reported that time discounting was correlated with greater weight loss and lower energy intake in response to weight regulation interventions 20, 62, 63. Best *et al.* (2012) study whether time discounting correlates with successful weight loss among 241 overweight children (aged 7–12 years) enrolled in an obesity programme, in which children were randomized into one of three interventions: dietary change, physical activity and self‐control promotion. Further, the authors test for an interaction with the ‘relative reinforcing value of food’. Reinforcement refers to the propensity of a stimulus to increase the intensity of behaviour that precedes it, such as the actions that an addict might engage in to obtain drugs 64. In this case, it captures how hard children are prepared to work for food relative to money. Time discounting was the strongest predictor of weight loss by the end of treatment at 16 weeks, but only among children with high reinforcing values of food 62. Appelhans *et al.* (2013) test whether time discounting correlates with energy intake in a sample of obese and overweight women enrolled in a programme where subjects were advised how to make a daily calorie deficit in their diets. The authors report steeper delay discounting among those with higher energy intake, but only among those who ate ready‐to‐eat or away‐from‐home meals rather than home‐prepared meals 20. Weygandt *et al.* (2015) explore the neural correlations of delay discounting and body weight maintenance in 19 subjects a year after a 12‐week diet. The authors find that lower delay discounting was associated with greater success in weight maintenance 63.

### Discount rate measures

Three studies reported varying associations between time discounting and unhealthy diets or body weight according to the discount measure used. Lim *et al.* (2015) report no correlation between discount rates computed for monetary and weight loss rewards. Discount rates were correlated with attitudes towards obese persons. Higher discount rates for weight loss were associated with beliefs that obesity is under the obese persons' control, whilst higher discount rates for money were associated with explicitly positive attitudes towards obese persons 34. Rasmussen *et al.* (2010) report higher discount rates for hypothetical food in persons with higher percentage body fat. These findings did not extend to discounting for monetary rewards, and there was no association between either discount measure and BMI 36. Manwaring *et al.* (2011) also reported significant differences in discount rates for food but not monetary rewards between BED, obese, non‐obese and non‐BED groups 51.

## Discussion

Our systematic review highlights a number of important findings on the relationship between diets, body weight and time discounting. First, we found consistent evidence of higher time discount rates in persons consuming unhealthy diets and overweight and obese individuals. Second, the number of studies testing for discount rate modifiers was small, but a mindful eating workshop, a prospective imagery intervention and a reasoning task were all associated with reduced discount rates. Third, experimental studies indicate that higher time discounting is linked to less weight loss and higher energy intake in the context of weight loss interventions. Fourth, time discount rates measured using hypothetical and monetary discounting measures tended to find null results, whereas those using actual and food‐based rewards had stronger patterns.

Our review has important limitations. First, because of methodological variations across studies in measuring time discount rates and higher risk of overweight and obesity, it was not possible to perform a meta‐analysis or calculate pooled effect sizes. To address this limitation, we have structured the review by type of study, so permitting comparisons within and across study designs. Second, we identified a relative dearth of higher‐quality longitudinal studies and experimental designs. This may fail to account for potential bi‐directionality, whereby unhealthy diets high in sugar increase time discounting rather than vice versa. For example, unhealthy diets rich in sugary foods could increase discount rates, for example, by reducing the sensitivity of the brain's reward centre 17, 65, 66. Third, studies also strongly relied on convenience sampling, particularly of college‐age studies, that may limit external validity.

The systematic review highlights several important gaps in knowledge for future research. There were gaps in understanding how time discounting affects response to diet and weight loss interventions: two out of the three studies were restricted to children and women, and the third study had a small sample size (*n* = 19) that could account for null findings. Additionally, there is a need to better understand whether higher discount rates in individuals with higher BMIs and obesity are specific to food discounting or whether they also extend to money. As highlighted in our review, findings varied according to whether discounting of monetary or food rewards was being measured. Importantly, three studies that measured food and monetary rewards found significant results using food but not monetary discount measures. It is also possible that rewards are discounted more steeply than losses 67, yet none of the existing studies tested this potential asymmetry. There is a need for future research to better identify the specific types of discounting (risk vs. delay, gains vs. losses, food vs. money) that increase risks of unhealthy food consumption, overweight and obesity.

There is also potential confounding with other cognitive factors and preferences, such as risk aversion. If study participants view delayed rewards as less certain, then time discounting measures may actually capture risk aversion, also known as ‘probability discounting’. The four studies included in our review that measured probability discounting as well as delay discounting had contrasting results. Two reported higher probability discounting in individuals who were obese, had higher BMIs and with higher per cent body fat 36, 51; but another two studies documented no group differences in probability discount rates 42, 53.

Finally, none of the studies we reviewed analysed the social, life‐course origins of discount rates in order to test whether these explain the social patterning of unhealthy diets and obesity 15, 68, 69. Sociological research indicates the importance of social and environmental factors for both diets and discounting, for example, via their effects on cognitive function. Thus, poor cognitive function is linked to higher discounting and is hampered by stress, leading Bickel and colleagues, in a recent review, to highlight the potential role of stress‐related socioeconomic factors such as poverty in influencing unhealthy behaviour via increased discounting 70. Yet research into the life‐course determinants of cognitive function highlights the potential contribution of language development, home learning environments, parenting style and beliefs and health (maternal health, birth weight and breastfeeding) 71, 72, 73. The social patterning of these environmental factors corresponds to social gradients in overweight and obesity suggesting that all could contribute to unhealthy diets and obesity via their knock‐on effects on time discounting. Future research should address this limitation in the literature by identifying the social determinants of time discounting. This evidence is needed to inform policy on upstream interventions to mitigate the risk of overweight and obesity, for example, by understanding better the observation of an association between discount rates and density of fast food outlets by DeVoe *et al.* 48. It may also identify the social groups for whom food and weight loss interventions may be least effective.

Notwithstanding these limitations, our review found moderate evidence that higher time discounting is positively associated with obesity and unhealthy diets. Our findings have important implications for policies aimed at curtailing obesity and encouraging healthy eating. These are particularly relevant to the UK's Behavioural Insights Team who are experimenting with health‐promoting interventions that are grounded in insights from behavioural economics 74, 75, 76 and the US government's Executive Order calling for greater use of insights from behavioural science in public policy 77. Effective policies are based on an understanding of the factors that influence or constrain the choices that people make 3. Thus, interventions such as financial incentives for healthy eating or weight loss that assume a flat rate of discounting may fail to capture perceptions of costs and benefits over time of overweight persons and persons with unhealthy diets and therefore risk being ineffective 78. Higher discount rates may act as a ‘trans‐disease process’, playing a role in several risky health behaviours including hazardous alcohol consumption, tobacco use and physical inactivity 79. If possible to identify clinical or policy measures shaping discount rates, it may yield a powerful means to reduce multiple sources of obesity risk simultaneously 80.

## Conflict of interest statement

No conflict of interest was declared.

## Supporting information

Web Appendix 1 – PRISMA checklist of items to include when reporting a systematic review or meta‐analysis (Moher *et al.* 2009).

Supporting info item
